# Synthetic peptides mimicking lipopolysaccharide as a potential vaccine candidates against *Vibrio cholerae* serogroup O1

**Published:** 2017-08

**Authors:** Fatemeh Mohammad Pour Ghazi, Seyed Latif Mousavi Gargari

**Affiliations:** Department of Biology, Faculty of Basic Sciences, Shahed University, Tehran, Iran

**Keywords:** Immunization, Peptides, *Vibrio cholerae*, Mimotope, LPS

## Abstract

**Background and Objectives:**

Cholera is a life-threatening diarrhea caused mainly by Gram-negative marine habitant *Vibrio cholerae* serogroup O1. Cholera vaccination is limited mainly to developed countries, due to the cumbersome and expensive task of vaccine production. In the present work, the aim was to study the immunogenicity of the synthetic mimotopes through two different routes of injection and oral administration. Lipopolysaccharide (LPS) is one of the immunogenic components in Gram-negative bacteria, which cannot be used as a vaccine candidate, due to its high toxic effect.

**Materials and Methods:**

Three phage-displayed selected peptides, with high affinity to anti-LPS VHH tested in our previous study, were chemically synthesized and used as a potential vaccine candidate. In order to enhance the antigenic properties and safe delivery, these peptides were conjugated to BSA as a carrier and encapsulated with PLGA. Peptides were injected intra-peritoneally or administered orally, alone or in combined form. Mice sera and feces were collected for assessment of humoral and mucosal antibody titers, respectively. ELISA plates were coated with mimotope conjugates and *V. cholerae*, *Shigella sonnei* and ETEC were used as target antigens. Antibody titer was measured by adding IgG and IgA as primary antibodies.

**Results:**

Mice receiving three selected synthetic peptide conjugates (individually or in combination) showed higher antibody titer compared to control groups. The mice immunized with synthetic peptides were protected against more than 15 LD50 of *V. cholerae.*

**Conclusion:**

These peptides are mimicking LPS and can potentially act as vaccine candidates against *V. cholerae.*

## INTRODUCTION

Cholera is an epidemic malignant infectious disease caused by curved bacillus; *V. cholerae*, a Gram-negative bacteria distributed in marine environment. This infection is mostly present in water supply environments by cholera patients. The main symptom of cholera is rice watery diarrhea, if left untreated could lead to severe dehydration and death in short period (1). In 2011, WHO reported 589,854 cholera cases with an increased death rate from 7543 to 7816 in 85 countries (2). *V. cholerae* is classified into 206 serogroups by the key antigen lipopolysaccharide (LPS). Serogroup O1 is the main infectious agent, responsible for all pandemics (3). The difference in the carbohydrate structure of O antigen in the LPS is the base of serological classification of *V. cholerae*. In this case, the serotype with A and B antigens is known as Ogawa and the one expressing A and C antigens is called Innaba (4, 5). Active vaccination is an additional and effective approach to prevent widespread bacterial infections, creating a passive immune response (1, 6). LPS, which is one of the main pathogen-associated molecular patterns (PAMPs) seems to be well suited to immune system induction. LPS has an invariant structure among all serotypes of vibrio and semi conserved structure in Gram-negative bacteria. The immunity against this component may be protective for a wide range of Gram- negative bacteria. As a unique component of bacterial cell wall, LPS can lead to better antigen recognition by immune system. The antigenic part of LPS is the carbohydrate chain which is named O antigen (7).

Most of our knowledge about carbohydrate immune response comes from microbiological studies about bacterial pathogenicity. Polysaccharides (PSs) are known as thymus independent antigens. Although their immunogenicity depends on late developing B-cell activity, they do not require mature T-cells to induce humoral response in the immune system (7). PSs are only immunogenic in adults, non-immunogenic or poorly immunogenic in newborn children and immune-deficient individuals. The use of pure PSs in the form of vaccine is limited due to their inefficiency in eliciting real immune responses in high-risk individuals. The complicated aspects of PS carrier conjugates made an increased interest for designing an alternative vaccination strategies using molecular mimicry. Peptides can be used as a substitution of a natural PS antigen due to less complicated structure compared to PSs with lower molecular weight and thymus-dependent immune nature (8, 9). Phage display is a technique used to express exogenous peptides and present them on the bacteriophage surface (10). The advantage of peptides as mimotopes is their linear sequence and short size, making their synthesis easy, in comparison to the originally conformational epitopes. In addition, peptides are shown to be able to raise a protective anti pattern-PSs antibodies (11, 12). Sufficient evidences are not available about the correlation of structural mimicry in peptides and the mechanism of inducing anti-mimotope antibodies responding to the pattern polysaccharide. Despite the aforementioned facts, still many peptides exist that are defined as an immune response inducers against particular bacterial PSs (11, 12).

In our previous report (13), we directly immunized mice with recombinant phages, harboring foreign peptide mimitopes. In the present work, in order to develop an effective anti-cholera vaccine, we employed synthetic phage-displayed peptides for animal immunization, followed by analysis of elicited immune responses. The present study intended to introduce three mimotopes individually and in combinations, as a real step in development of peptide based vaccine techniques to prevent cholera.

## MATERIALS AND METHODS

### Peptide synthesis.

The phagemids containing three high-affinity displayed peptides from our previous report (13) named here as A12X, A9X and A1X, were used to identify the peptide genes sequences. The three peptides were synthesized using these sequences as the patterns. The sequences are exhibited in Table 1.

**Table 1.** Vaccination protocol of different mice groups.

**Route**	Mice*		

	Injection**	**oral**	

		PLGA encapsulated conjugates	Non-polymerized conjugates
Experimental groups based on peptide names (5mice/group)	A1X-BSA	A1X-BSA	A1X-BSA
	A9X-BSA	A9X-BSA	A9X-BSA
	A12X-BSA	A12X-BSA	A12X-BSA
	Mix of three+-BSA	Mix of three-BSA	Mix of three-BSA
	Control: BSA	Control: encapsulated BSA	Control: BSA

* Eight to nine week-old female mice

** injected intraperitoneally with 150 μg of three peptide conjugates

+ Mixture of three peptides

### Peptide-carrier conjugation.

Bovine Serum Albumin (BSA) was selected as the carrier and the conjugation procedure was carried out by dissolving 100 mg BSA in 2 ml double-distilled water (DDW). Then, 10 mg peptide was added to the suspension, followed by addition of glutaraldehyde to a final concentration of 0.1%. The pH was adjusted at 7.8 and the solution was incubated for 8–12 h at 4°C with gentle rotation. A tiny pinch of NaBH_4_ was added to inactivate remaining glutaraldehyde. The yield of conjugation was assayed by spectrophotometer and the conjugated peptides were stored at −20°C, until further use.

### Polymerization of peptide conjugates.

For polymerization, 1 mg conjugated peptide is added to 2 ml PLGA suspension in methylene chloride (1/25 v/v%), using vigorous shaking. After adding 30 ml polyvinyl alcohol (2/5 v/v%), the solution was centrifuged at 7000 × g for 5 min and the precipitated particles were collected and washed three times with DDW.

### LD50 assay.

Eighteen pups of five days old NIH mice were isolated from their germ free dams and divided into three groups. Mice groups 1–3 were pipette fed with 10^6^, 10^7^ and 10^8^ CFUs of *V. cholerae*, O1, ATCC 39315, respectively and the death rate was monitored for 48 h. The bacterial dose in which half of the mice were left alive, considered as LD50, as described by Sawasvirojwong et al. (14).

### Immunization.

Eight to nine week-old female mice were divided into three main groups according to route and form of antigen used for immunization. Each group was further divided into five subgroups, each having five mice. Each of the three subgroup, in group one was injected intraperitoneally with 150 μg of three peptide conjugates individually and the fourth one received a mixture of the three peptides. The fifth subgroup was kept as a control, receiving BSA. Three doses of antigens were given to animals on days 0, 14 and 28. The main groups 2 and 3 were also divided into subgroups and orally fed with polymerized and non-polymerized peptide conjugates, respectively. The pattern of immunizations was similar to that of main group one. Encapsulated BSA and BSA alone were considered as controls. The whole immunization process is summarized in Table 2.

**Table 2.** The DNA and Amino Acid sequences of three phage displayed peptides presented on PIII protein of M13: there is no distinct repetition in amino acid contain in each peptide

**phage**	**DNA sequence**	**Peptides sequence**
A1X	5`:TTACCATCGGCCGGGCGTGGTGTCTGTTATGAAGCG	LPSAGRGVCYEA
A9X	5`: CAGCACCTCAATAGTATTTTGCTTGTAACGAAGGG	QHLNSILLVTK
A12X	5`: TTATCCTCACGGCTATGTTGCCTTTGGACGATGGCT	YPHGYVAFGRW

### Sample collection and LD50 estimation.

Ten days after the third immunization, blood samples from all mice groups were collected and the IgG titer was estimated. The feces were also collected for IgA titer testing. Twenty-one days after the third injection/feeding, all animals were treated with 100 μl of streptomycin (15 μg/ml), in order to inhibit gut flora manifestation. Different folds of LD50 (1, 5, 10 and 15) from *V. cholerae* serogroup O1 were prepared in 100 μl normal saline and fed to mice groups. The control group was fed with 100 μl normal saline. About 48 h after inoculation, the survived mice were counted.

### Serum ELISA.

The possible immune response against the selected peptide conjugates and their combination was evaluated, using ELISA. Immunized female mice sera and anti-sheep antibody conjugated with HRP were used as primary and secondary antibodies, respectively.

In the first ELISA, each peptide and the associated mixture were coated as target antigens on ELISA plates and the serum of each group was added to appropriate wells. In order to find out the affinity of the mice sera to the LPS of other Gram-negative bacteria, the second ELISA was performed by coating the ETEC (ATCC35401) and *S. sonnei* (ATCC9290) as targets.

A clinical isolate of *V. cholerae* from Iranian patient were used in the 3^rd^ ELISA.

### Fecal ELISA.

Three ELISA assays were also performed to detect the fecal IgA titer, in orally treated mice groups. The fecal IgA was extracted from mice feces by salt extraction method. Each conjugated antigen either alone or in combination with others were coated on the plates as the targets. The extracted IgA from mice feces orally fed with polymerized and non-polymerized antigens were used as primary antibody and anti-IgA anti-sheep antibody conjugated with HRP was used as the secondary antibody.

ELISA with the vibrio clinical strain and the other two above-mentioned Gram-negative bacteria was also carried out with IgA, as described for IgG.

## RESULTS

### Peptide synthesis.

The DNA fragments coding for three selected peptides on phagemids were sequenced and the peptides were synthesized accordingly by Biomatik, Canada. The sequence analysis of the peptides are given in Table 2.

### LD50.

Five to 7 days-old infants were fed with different amounts of bacteria to find out the LD50 for *V. cholerae*. The amount of 10^7^ CFUs was selected as LD50. Challenge experiments were based on LD50 fold.

### Immunization.

**Fig. 1.**
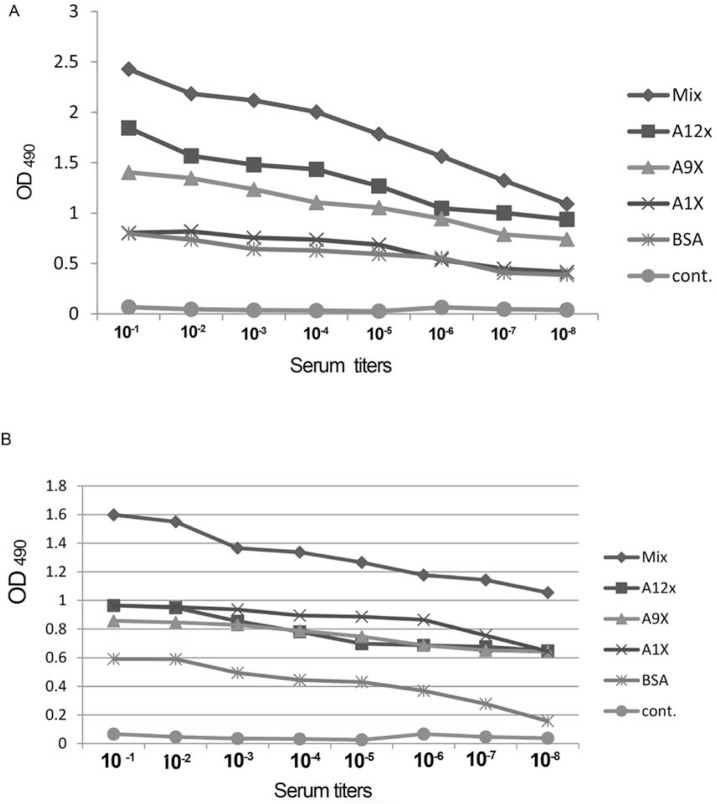
IgG titer against peptide conjugates, A: ELISA estimation of antibody titer of mice sera. Higher IgG titer is obtained against the combined form of mimotopes. B: Elicited serum responses against the polymerized peptides. Higher titer of IgG against the mixture of three polymerized form of peptides.

The sera obtained from immunized and control mice groups were used to evaluate the mimotope specific antibody titers. Sera against BSA also was tested to rule out any possible cross-reactivity of sera against BSA. The mimotope specific IgG titer was 33% higher than the control group. Our ELISA results indicated that, all three BSA conjugated peptides, either alone or in combinations, administered orally or by injection were immunogenic. No significant IgG response was elicited against the naked peptide conjugates fed orally. The antibody raised against the mixture of three synthetic peptides was not significantly higher than those administered alone (Fig. 1 A and B).

**Fig. 2.**
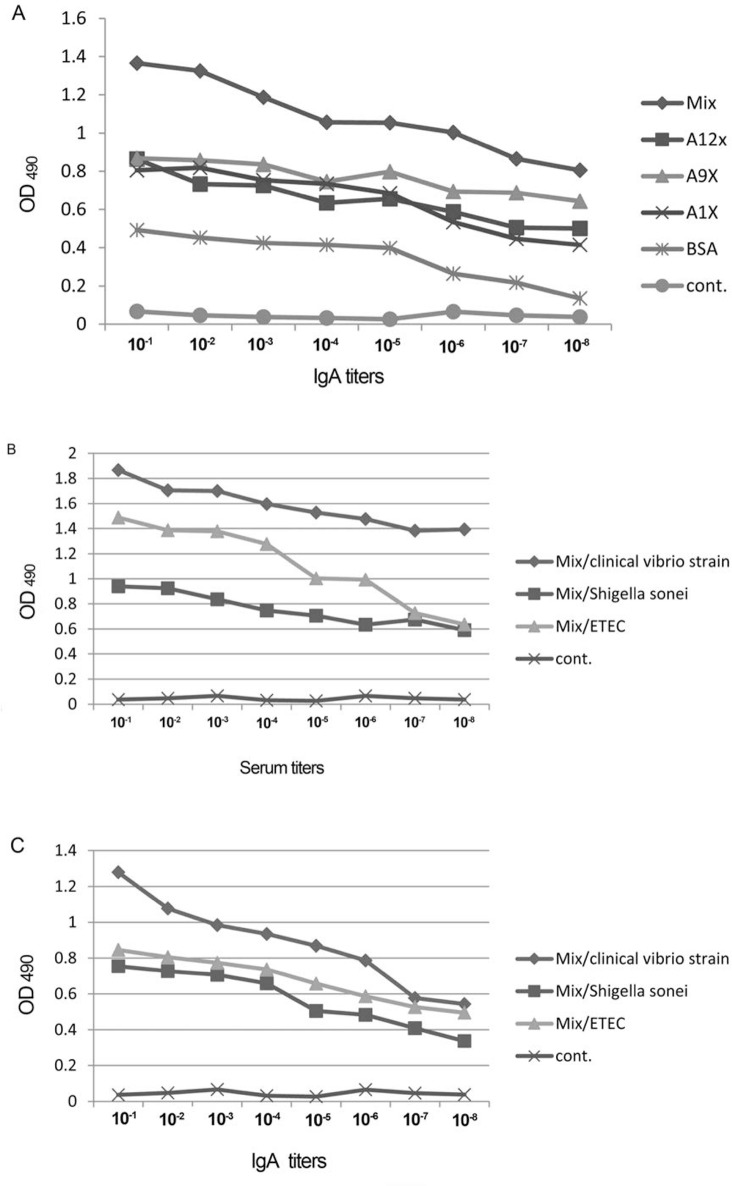
Elicited mucosal antibody. A: The IgA titer against the polymerized peptide conjugates and their combined forms orally given to mice B: ELISA results of sera IgG raised against injection of peptide conjugates using clinical strain of *V. cholera*, *S. sonnei* and ETEC as targets. C: ELISA results of IgA against orall administration of polymerized conjugates using *V. cholerae*, *S. sonnei* and ETEC as targets on ELISA plates.

The mucosal antibody response was also estimated by ELISA. Non-polymerized peptides did not show significant mucosal antibody response compared to the control group. In contrary to non-polymerized mimotope conjugates, the encapsulated-epitopes could raise the antibody titer significantly. The higher mucosal antibody was observed against the combined form of the three mimotopes compared to each one applied individually. The results are exhibited in Fig. 2A.

The affinity of IgG to clinical strain of *V. cholerae* was also estimated. The sera obtained from the mice injected with the mixture of three synthetic mimotopes showed higher affinity to the clinical strain, compared with each one applied individually.

IgG and IgA, both could detect *S. sonnei* and ETEC in cell ELISA but the OD_490_ obtained was lower than that of vibrio. Similar results were observed with fecal IgA (Fig. 2B and 2C).

### Protection.

In order to see the protectivity of synthetic peptides, all mice groups either injected or orally fed, were challenged by 5, 10, 15 folds of LPS of *V. cholerae* serogroup O1. Mice groups either injected with mixture of synthetic peptides or fed with encapsulated forms of the same peptides, could tolerate up to 15 LD50 of *V. cholerae* serogroup O1 for 72 h. Whereas mice orally administered with mixture of unpolymerised peptides, died with the same treatment of bacteria.

## DISCUSSION

The correlation between PAMPs and induction of innate immunity in human is approved before and LPS is of the main cell wall structure of Gram-negative bacteria known as a PAMP (6). LPS can induce the immune system in the form of detoxified component, but it needs suitable carrier to become immunogen. In our previous report, we introduced three peptides displayed on the phage M13 surface, as LPS mimicking sequences eliciting immune responses. Although phage particles as carriers successfully induced the immune response, but there are certain limitations on using phage particles directly as a vaccine. These includes: a) finding suitable host range for this type of phage libraries; b) high risk of administration of phage particles due to high amounts of exo and endotoxins production; c) phage inactivation as a result of sterilization of phage particles using current methods during vaccine preparation (15); d) longevity and visibility of phage particles in filtering organ of immune system such as spleen (16). Therefore, in the present study, we decided to chemically synthesize the mimotopes and use a protein carrier in order to induce a protective antibody. Attaching peptides to a protein carrier can be a simple and useful strategy for optimizing the selected peptides immunity (17, 18). BSA was preferred to use as a carrier due to its natural immunogenicity and simple conjugation process where each BSA molecule attaches to about 60 peptides. BSA is also recognized by T-cells due to its large size and has probably many epitopes presented on major histocompatibility complex (MHC) molecules (19). Three peptides were conjugated to BSA, each pep-BSA and their mixture was administered separately by oral gavage and injection. The results of serum ELISA show that all three pep-BSA conjugates and the mixture of three were immunogenic. It seems that BSA as a carrier was successful in inducing immune response against peptides *in vivo* (19, 20).

The synthetic vibrio vaccines used in this study have two important advantages: first there is no need for adjuvant due to their conjugation with BSA; and the second is the ease of application because poly ethylene glycol (PEG) is not used for purification, before use. The solution containing PEG and NaCl is generally used to purify the phage particle and to eliminate LPS (13).

Unlike the weak antibody response for *Brucella* LPS mimotopes and moderate affinity of *Shigella flexneri* mimics LPS (21), our synthetic vibrian LPS mimiotopes could significantly induce antibody titer. The IgG titer was higher in injected mice compared to mice that were orally-fed with peptides.

Higher titer of antibody against the combined form of three synthetic peptides compared to individually injected one, could be a consequence of combination of cellular and humoral response as more extended epitopes length will be recognized by both T and B-cells (13). After detecting each peptide as a distinct epitope by separate B and T cells, antibodies may be raised. Both of these immune response patterns can lead to an increased antibody titer. We hypothesize that longer peptides would induce a stronger responses due to the support of stronger T-cell epitope recognition. In other words, three peptides with high affinity to VHH, may mimic the epitopes of LPS and lead to higher titer of raised antibody in comparison with each selected peptide. Similar results were obtained when tested with phage-displayed peptide (13).

No significant mucosal antibody response against non-polymerized peptides probably is due to the degradation of mimotopes by the gastro-intestinal acid and enzymes like peptidase and proteinase (22). In addition, immune system was facing with the bulk entry of mimotopes in non-polymerized form. We speculate that facing a mass antigen would cause tolerance in mucosal immunity leading to lack of response from immune system (22). Therefore we did not observe significant increase in the antibody titers (IgG and IgA) when non-polymerised peptides were fed orally. A response of encapsulated-epitopes further explains that PLGA-encapsulated conjugates could aid the immune system to recognize the epitopes, followed by slow releasing of the antigens in the small intestine, with no tolerance and stomach degradation. The similarity of ELISA results with clinical vibrian strains as well as with *Shigella sonei* and ETEC could be attributed to the similarity of LPS structure in Gram-negative bacterial surface. Therefore, LPS mimotope vaccine might inhibit the other Gram-negative bacteria colonization.

## CONCLUSION

Synthetic peptides such as mimotopes have more advantages over the other current methods. Immune regulatory and infection inhibitory, characteristics of phage-displayed mimotopes would be useful in improving phage-based protocols of epidemic-infection therapy. Since the results of this research is approximately similar to that of our previous report where we used whole phage particle as a vaccine candidate (13), herein we suggest the use of synthetic mimotopes instead of whole phage particle, due to ease of production and use of BSA conjugates and possible afore-mentioned adverse effects of phages. Although for cholera as an enteric infection, oral vaccination is advised, but our findings showed that both oral and injective administration with mimotopes seems to be effective in eliciting protective immune responses (23).
